# Functional Characterization of Lysophospholipids by Proteomic and Lipidomic Analysis of Fibroblast-like Synoviocytes

**DOI:** 10.3390/cells12131743

**Published:** 2023-06-29

**Authors:** Thomas Timm, Christiane Hild, Gerhard Liebisch, Markus Rickert, Guenter Lochnit, Juergen Steinmeyer

**Affiliations:** 1 Protein Analytics Group, Institute of Biochemistry, Justus Liebig University Giessen, 35392 Giessen, Germany; 2 Laboratory for Experimental Orthopedics, Department of Orthopedics, Justus Liebig University Giessen, 35392 Giessen, Germany; 3 Department for Clinical Chemistry and Laboratory Medicine, University Hospital Regensburg, 93053 Regensburg, Germany

**Keywords:** lysophosphatidylcholine, lysophospholipid, proteomics, lipidomics, phospholipids, biosynthesis, lipids, efflux, inflammation, orbitrap, functions, osteoarthritis, fibroblast-like synoviocytes

## Abstract

Synovial fluid (SF) from human knee joints with osteoarthritis (OA) has elevated levels of lysophosphatidylcholine (LPC) species, but their functional role is not well understood. This in vitro study was designed to test the hypothesis that various LPCs found elevated in OA SF and their metabolites, lysophosphatidic acids (LPAs), modulate the abundance of proteins and phospholipids (PLs) in human fibroblast-like synoviocytes (FLSs), with even minute chemical variations in lysophospholipids determining the extent of regulation. Cultured FLSs (*n* = 5–7) were treated with one of the LPC species, LPA species, IL-1β, or a vehicle. Tandem mass tag peptide labeling coupled with LC-MS/MS/MS was performed to quantify proteins. The expression of mRNA from regulated proteins was analyzed using RT-PCR. PL synthesis was determined via ESI-MS/MS, and the release of radiolabeled PLs was determined by means of liquid scintillation counting. In total, 3960 proteins were quantified using multiplexed MS, of which 119, 8, and 3 were significantly and reproducibly regulated by IL-1β, LPC 16:0, and LPC 18:0, respectively. LPC 16:0 significantly inhibited the release of PLs and the synthesis of phosphatidylcholine, LPC, and sphingomyelin. Neither LPC metabolite—LPA 16:0 nor LPA 18:0—had any reproducible effect on the levels of each protein. In conclusion, small chemical variations in LPC species can result in the significantly altered expression and secretion of proteins and PLs from FLSs. IL-1β influenced all proteins that were reproducibly regulated by LPC 16:0. LPC species are likely to modulate FLS protein expression only in more advanced OA stages with low IL-1β levels. None of the eight proteins being significantly regulated by LPC 16:0 have been previously reported in OA. However, our in vitro findings show that the CD81 antigen, calumenin, and B4E2C1 are promising candidates for further study, focusing in particular on their potential ability to modulate inflammatory and catabolic mechanisms.

## 1. Introduction

Osteoarthritis (OA) is a chronic musculoskeletal disease that affects the entire joint, characterized by the progressive destruction of articular cartilage. Compared with normal subjects, the profile of joint lubricants in the synovial fluid (SF) of OA patients is altered, harboring elevated levels of phospholipids (PLs), hyaluronan, and lubricin [1]. Our previous analysis showed that phosphatidylcholine (PC) and lysophosphatidylcholine (LPC) species account for approximately 67% and 10% of all PLs in human SF, respectively [2,3]. Compared with healthy SF, the levels of both PL classes are significantly higher in OA in humans and a canine model of OA, peaking during late-stage OA [2,4].

PC and LPC species are derived from plasma through fenestrated synovial capillaries and actively secreting fibroblast-like type-B synoviocytes (FLSs) [5]. Several reports have ascribed pathophysiological functions to insulin-like growth factor-1 (IGF-I), transforming growth factor-β1 (TGF-β1), and interleukin-1β (IL-1β) during OA, and elevated levels of IGF-1 and IL-1β have been detected in SF in human OA [6,7,8,9]. IGF-1, TGF-β1, and leptin stimulate FLSs and chondrocytes in human OA to synthesize elevated amounts of PC and LPC [10,11]. Levels of the proinflammatory cytokine IL-1β in SF peak only in the early stages of knee OA with a radiological Kellgren–Lawrence (KL) score of I, whereas in more advanced stages, with a KL grade of II–IV, its concentrations are four–five-fold lower [8]. In addition, Ning et al. [9] reported significantly lower concentrations of IL-1β in patients with a KL score of IV compared with those of early-stage knee OA. Recently, we found that IL-1β stimulates the release of PC from OA FLSs through the upregulation of membrane-associated ABCA1 transporters [5].

In mammalian cells, LPC species function as potent signaling molecules, and their levels need to be strictly controlled. LPC is generated from PC via hydrolysis through phospholipase A2, the levels of which, including phospholipase A2 group V, are elevated in human OA cartilage and synovial membrane compared to those of the control [12,13]. LPC species can reenter the Lands cycle to be reacylated by lysophospholipid acyltransferases, deacylated further primarily by lysophospholipase A1 and A2, or hydrolyzed by lysophospholipase D (also called autotaxin), generating choline and biologically active lysophosphatidic acid (LPA) species. LPAs mediate neuropathic pain in mice and are involved in inflammation [14,15]. Autotaxin is also expressed in OA FLSs, and both plasma and SF autotaxin are associated with the radiographic severity of OA [16,17]. Bioactive LPAs signal through six G-protein-coupled receptors, LPA_1–6_ [18], and have been suggested to contribute to or cause some of the effects of LPC [19,20].

The receptors for LPC species are the G-protein-coupled receptor GPR132 (G2A) and GPR4 and Toll-like receptors 2 and 4. Several pathways are activated after stimulation with LPC, including NF-κB, p38 MAPK, and JNK, affecting a variety of functions, such as the release of inflammatory factors, oxidative stress, and apoptosis [20,21].

It is assumed that biological information is stored in the chemical composition and consequent three-dimensional structure of LPC species. Several studies have compared the function of saturated and unsaturated LPC species in various cell types and diseases [21]. In endothelial cells, LPC 16:0 and LPC 20:4 induce the marked mRNA and protein expression of cyclooxygenase-2, whereas LPC 18:1 fails to upregulate this protein [22]. The stimulatory effect of LPCs on IL-1β expression in monocytes has been shown to depend on the acyl chain length and degree of saturation [23]. For instance, LPCs with an acyl chain of less than 16 have no effect on IL-1β synthesis, whereas LPC 18:0 enhances it more than the monounsaturated LPC 18:1 [23]. Moreover, among the saturated (C6-C18) and monounsaturated acyl (LPC 18:1) LPCs that have been tested in intraperitoneal injections in mice, LPC 16:0 has the strongest proinflammatory activity. This was antagonized by the intraperitoneal injection of polyunsaturated LPC 20:4 and LPC 20:6, indicating that the acyl chain length and degree of saturation influence the control of inflammation [24].

However, the functions of LPC species in articular joints and during OA remain largely unknown. Recently, the ratio of LPC 18:2 to PC 44:3 in serum was reported to be associated with an increased risk of a cartilage volume loss in OA knees [13]. Moreover, based on a 10-year follow-up study, the LPC:PC ratio was proposed as a valuable metabolic marker that predicts advanced knee OA [25]. The intraarticular injection of LPC 16:0 in mice induces chronic pain through acid-sensing ion channel 3 and anxiety-like behavior but does not cause inflammation, peripheral nerve sprouting, or bone alterations [26]. Interestingly, the same investigators found significant correlations between the LPC 16:0 concentration in the synovial fluid of OA patients and global pain intensity and pain intensity in the affected knee, as measured by a visual analogue scale (VAS), which might be attributed to the above mechanism [26].

Collectively, these data on the various—at times differing or even opposing—effects of individual LPC species in healthy individuals and patients with OA are limited. To this end, modern lipidomic and proteomic analyses might be able to provide a significant breakthrough in our understanding of the complex functions of and between LPC species. Thus, we applied a novel multiplex proteomic technique, using isobaric tandem mass tags, to perform a simultaneous and semiquantitative mass spectrometry (MS) analysis of multiple samples. The aims of our proteomic and lipidomic study were to identify and discriminate the various effects of three LPC species that differed only slightly in their chemical composition and distinguish their effects from those that were induced by their corresponding LPA metabolites and by the proinflammatory cytokine IL-1β.

## 2. Materials and Methods

### 2.1. Materials

All reagents were purchased from Sigma (Deisenhofen, Germany) unless otherwise noted. DMEM, choline-depleted phenol red-free DMEM, 1M HEPES solution, and penicillin/streptomycin solution were obtained from PAN Biotech (Aidenbach, Germany). CHAPS was acquired from Roth (Karlsruhe, Germany), and DTT and formic acid were purchased from Fluka (Seelze, Germany). IPG buffer was obtained from GE Healthcare (Freiburg, Germany), trypsin (TrypsinGold™) was purchased from Promega (Madison, WI, USA), the Pierce™ BCA Protein Assay Kit was acquired from Thermo Fisher (Waltham, MA, USA), and [^3^H]-choline chloride was obtained from PerkinElmer (Rodgau, Germany). LPC 16:0, LPC 18:0, LPC18:1, LPA 16:0, and LPA 18:0 were purchased from Avanti Polar Lipids (Alabaster, AL, USA), and [D9]-choline and [D4]-ethanolamine were from Cambridge Isotope Laboratories (Tewksbury, MA, USA).

Before being added to the cell culture, LPC 16:0 and LPC 18:0 were each dissolved in a methanol:water (95:5) mixture using a glass vial, warmed slightly, sonicated, and aliquoted. The methanol:water mixture was dried up by using a nitrogen blowdown evaporator. Aliquots were frozen at −20 °C. LPC 18:1 was dissolved in chloroform using a glass vial, sonicated, and aliquoted. The solvent was removed using a nitrogen blowdown evaporator, and the dried aliquots were stored at −20 °C. Lipid films of all LPC species were dissolved in serum-reduced DMEM (5% FBS), which was also added as a vehicle to untreated controls in the same volume as LPC species. The two LPA species were each dissolved in a glass vial with 95% ethanol at room temperature, vortexed, sonicated, heated to 37 °C, and frozen in aliquots with a layer of argon at −20 °C. As a vehicle for the untreated controls, we used 95% ethanol, which was added to a well of a 6-well culture plate with the same volume as the LPA species.

### 2.2. Specimen Selection for Isolation of FLSs

FLSs were isolated from the synovial membranes of OA patients. Randomly chosen patients donated synovial tissue during knee replacement surgery at the Department of Orthopedics (Giessen), University Hospital Giessen and Marburg (Germany). All procedures in this study that involved patients were conducted as per the Declaration of Helsinki. Approval by the ethics committee of the Faculty of Medicine of the Justus Liebig University Giessen was obtained. All patients provided written informed consent to donate samples for research and publish any resulting data. No individual patient can be identified from the data in this paper.

The effects of lipid species on the FLS proteome were not examined in cells that were derived from excluded patients, with the exclusion criteria as follows [5,10]: RA; gout; trauma; joint infection or other joint disease; surgery, including knee joint surgery, within 3 and 6 months before the start of the study, respectively; a serious illness, such as HIV; tumors near the knee; severe disease of the kidney or liver; autoimmune disease; drug abuse; or use of corticosteroids, hyaluronan, or immunosuppressants in the 6 months before the start of the study.

### 2.3. Culture of FLSs

Human FLSs were isolated from the synovial tissue of OA knee joints as described [27]. Briefly, FLSs were propagated until passage 3–4 in a humidified 10% CO_2_ atmosphere at 37 °C using DMEM that was supplemented with 1.0 g/L glucose, 584 mg/L L-glutamine, 10% fetal bovine serum, 10 mM HEPES, 100 U/mL penicillin, and 0.1 mg/mL streptomycin. We verified that there was no mycoplasma infection using the I/C PCR mycoplasma test kit (PromoCell, Heidelberg, Germany).

Before being treated (see below), confluent FLSs were assessed for purity using FACS on a BD FACSCANTO II (Becton Dickinson, Heidelberg, Germany) as described [5,10]. Briefly, 1 well with cultured FLSs per patient was trypsinized and incubated with APC-labeled anti-human CD90 (clone 5E10) and PE-labeled anti-human CD45 (clone 2D1) (both from BioLegend, San Diego, CA, USA). As a result, 95.3% ± 4.9% of cultured FLSs stained positive for fibroblast-specific CD90 but negative for CD45.

### 2.4. Treatment of Cultured FLSs for Proteomic and mRNA Analysis

To analyze the proteome of FLS extracts, FLSs from passage 4 were first seeded into 6-well plates at a density of 80,000 per well. Cells were grown until confluence, with the media changed every 2–3 days using 4 mL of supplemented DMEM, as described above. Confluent FLSs were analyzed by FACS to verify their purity, as outlined above.

Confluent FLS received 2 mL of serum-reduced DMEM (5% FBS) for 48 h supplemented as described above, this time containing an additional 4 mg/L of folic acid, and the medium was changed after 24 h. Again, each well received 2 mL of the same serum-reduced DMEM (5% FBS) and one of the five lipid species, either 20 µM of LPC 16:0, LPC 18:0, or LPC 18:1 or 10 µM LPA 16:0 or LPA 18:0. Additional wells with confluent FLS and media received 1.0 ng/mL IL-1β or 20 µM LPC 16:0 together with 1 ng/mL IL-1β, whereas the corresponding controls received the vehicle. FLSs for proteomic analyses were cultured separately from those for mRNA expression analyses. After 48 h, the medium was removed, and a mycoplasma test was performed as described above. The cells were washed twice with 2 mL of ice-cold PBS. For subsequent mRNA expression analysis, washed FLSs were lysed by adding 0.35 mL of RP1 buffer (NucleoSpin^®^ RNA/Protein kit) and 3.5 μL of β-mercaptoethanol per well as per the manufacturer’s instructions (Macherey-Nagel, Düren, Germany) and sonicated. The extracts were snap-frozen in liquid nitrogen and stored at −86 °C.

For subsequent proteomic analysis, the remaining LPC-treated, LPA-treated, IL-1β-treated, and untreated FLSs of the same biological replicates, cultured in separate wells, were washed and dissolved in 0.5 mL of lysis buffer (6 M urea, 2 M thiourea, 4% CHAPS, 30 mM DTT, 2% IPG-buffer, pH of 3–10) per well with a pH of 7.0. The FLSs were detached from the bottom with a cell scraper and lysed in 0.5 mL of lysis buffer, and the extracts were sonicated and stored at −86 °C.

The experiment was repeated 5 times using FLSs that were donated from 6 OA patients during knee replacement surgery (age 69.7 ± 12.9 years, BMI 29.4 ± 4.2 kg/m^2^, CRP 3.1 ± 2.5 mg/mL, 2 males, 4 females). Some patients had comorbidities, including arterial hypertension (*n* = 4), hypothyroidism (*n* = 3), hyperlipidemia (*n* = 2), and fibromyalgia (*n* = 1). These 6 biological replicates of each treated and untreated FLS culture were analyzed twice, generating proteomic data from 12 replicate samples per treatment and their corresponding controls.

### 2.5. Protein Isolation and TMT Labeling

The cellular protein content of lysed FLS extracts was quantified using the 2-D Quant Kit (GE Healthcare, Freiburg, Germany) according to the manufacturer’s instructions. Approximately 25 µg of extracted proteins in each lysed cell culture was digested with trypsin (TrypsinGold^TM^, Promega, Madison, WI, USA) at a protein:enzyme ratio of 50:1 following the filter-aided sample preparation (FASP) protocol [28]. The tryptic peptides were then desalted using Chromabond C18ec cartridges (100 mg/1 mL; Macherey-Nagel, Düren, Germany), lyophilized overnight, redissolved in 50 µL 50 mM HEPES with a pH of 8.5, and quantified on a NanoDrop 2000 (Thermo Scientific, Waltham, MA, USA).

For each of the 6 biological replicates, 5 µg of peptides of the 9 individual treatments per biological replicate were labeled separately using 100 µg of tandem mass tag (TMT) labeling reagent (TMT10plex Isobaric Label Reagent Set; Thermo Scientific, MA, USA) according to the manufacturer’s instructions. The isobaric compounds in the Reagent Set were designated to the samples as follows: reporter ions at 126—untreated control, 127N—LPC 16:0, 127C—LPC 18:0, 128N—LPC 18:1, 128C—ethanol vehicle-treated control, 129N—LPA 16:0, 129C—LPA 18:0, 130N—IL-1β, 130C—LPC 16:0 + IL-1β, and 131—pool of the 6 untreated controls. The pooled control sample was derived from the same 6 biological replicates and was used as a standard to compare with the labeled samples.

### 2.6. Peptide Fractionation, Liquid Chromatography, and LC-MS/MS/MS

The MS analysis was performed as described [29]. Briefly, to increase the number of peptides that were identified in the LC-ESI-MS analysis, the labeled peptide mixtures were fractionated using the High-pH Reversed-Phase Peptide Fractionation Kit (Pierce™, Thermo Fisher Scientific, Waltham, MA, USA), according to the manufacturer’s protocol, resulting in 8 fractions for each of the 6 biological replicates.

For the MS analysis, 1 µg of the sample from the fractionation was loaded onto a 50 cm µPAC™ C18 column (Pharma Fluidics, Gent, Belgium) in 0.1% formic acid at 35 °C. The peptides were eluted with a linear gradient of acetonitrile from 3% to 44% over 240 min, washed with 72% acetonitrile at a constant flow rate of 300 nL/min (Thermo Fisher Scientific™ UltiMate™ 3000 RSLC nano), and infused via an Advion TriVersa NanoMate (Advion BioSciences, Inc., New York, NY, USA) into an Orbitrap Eclipse Tribrid mass spectrometer (Thermo Fisher Scientific, Waltham, MA, USA). The MS instrument was operated in positive ionization mode with the spray voltage and source temperature of the NanoMate system set to 1.7 kV and 300 °C, respectively.

### 2.7. Protein Identification and Quantitation

The parameters for the protein identification and quantification were identical to those described earlier [29]. Briefly, proteomic data were acquired with Xcalibur 4.3.73.11 (Thermo Fisher Scientific, Waltham, MA, USA). The data from the 8 fractions were merged into 1 dataset per biological replicate, and the resulting 6 datasets were analyzed in Proteome Discoverer 2.5.0.400 (Thermo Fisher Scientific, Waltham, MA, USA). Sequest HT (Thermo Fisher Scientific, Waltham, MA, USA) was used to search against the UniProt_human database (v. 11 December 2019). A precursor ion mass tolerance of 10 ppm was used, and 1 missed cleavage was allowed. The fragment ion mass tolerance was set to 0.6 Da for the linear ion trap MS2 detection. The false discovery rate (FDR) for peptide identification was limited to 0.01 using a decoy database.

Identified proteins were accepted if they could be established with at least 1 unique identified peptide with a length of between 6 and 144 amino acids. TMT reporter ion values were quantified from MS3 scans with an integration tolerance of 20 ppm. The reporter ion values for the individual conditions were normalized to the TMT 131 channel, which was used as a common reference for all 6 biological replicates.

### 2.8. Analysis of mRNA Expression

After isolation of RNA according to the manufacturer’s instructions, the 260/280 nm ratios of the total extracted RNA samples were 1.97 ± 0.07, as determined on a NanoDrop™ 2000 (Thermo Fisher). The total RNA was then reverse-transcribed using the QuantiTect Reverse Transcription Kit (Qiagen, Hilden, Germany) as per the manufacturer’s instructions. The resulting cDNA was analyzed using QuantiNova LNA PCR assays (Qiagen) and the QuantiNova SYBR Green PCR kit (Qiagen) on a 7500 Fast Real-Time-PCR system (Applied Biosystems, Waltham, MA, USA) for the following genes: APOL2 (SBH0263823), CALM3 (SBH0063509), CALU (SBH0476886), CD81 (SBH0266209), HIST3H2A (SBH0235978), JAGN1 (SBH0184282), MCFD2 (SBH0188942), MT2A (SBH0576025), PLA2G4A (SBH0564600), SOD2 (SBH0210608), RPL27A (SBH0138340) SRM (SBH0333670), SSR4 (SBH0430063), TIMP1 (SBH0559541), TMED7 (SBH0099072), and TPM2 (SBH0143963). QuantiNova LNR PCR assays comprise primer pairs that are predesigned, bioinformatically validated, and specific for a target cDNA. Every biological replicate was examined in 2 technical replicates.

Using the serial dilution method [30], the efficiency of all primer pairs was 92% to 105% (98.8% ± 4.0%; *n* = 16). The 2^−ΔΔCt^ method was applied to determine the relative gene expression between treated versus untreated FLSs using GAPDH (SBH1220545) as the endogenous reference gene.

### 2.9. Treatment of FLSs to Study PL Release

We quantified the radiolabeled PLs that were released by FLSs during treatment, as described in [5]. Briefly, FLSs were cultured in 6-well plates using 2 mL choline-depleted, phenol-red-free DMEM that was supplemented as above. Confluent cells from passage 4 were analyzed by FACS to verify their purity, as described above, and labeled with 5 µCi/mL [^3^H]-choline chloride for 24 h. FLS were thoroughly washed to remove unincorporated isotopes, and FLSs were starved for 24 h with 2 mL serum-reduced DMEM (5% FBS) that was supplemented as described above. The media were changed, and cells received 20 µM of an LPC species, 10 µM of an LPA species, or vehicle as control for 48 h. After 24 h, the media with the treatments were changed. The media from the final 24 h of culture were used to determine the release of radiolabeled PLs from FLSs. This experiment was repeated 5 times using FLSs from 6 OA patients (age: 72.8 ± 8.1 years, BMI: 30.9 ± 2.4 kg/m^2^, CRP: 1.13 ± 1.38 mg/L, 2 males and 4 females). Some patients had comorbidities, including arterial hypertension (*n* = 5), hypothyroidism (*n* = 1), type II diabetes (*n* = 1), and hyperlipidemia (*n* = 3).

### 2.10. Quantification of Radiolabeled PLs

After treatment, the FLSs and media were collected separately. The cells were washed twice with ice-cold PBS and lysed with 0.1% sodium dodecyl sulfate (SDS). Lipids were extracted from the media and cell lysates using the Bligh and Dyer method [31]. The chloroform phase, containing the radiolabeled PLs, was mixed with liquid scintillation cocktail (Emulsifier-Safe™; Perkin Elmer), and radioactivity was measured in triplicate on a multipurpose scintillation counter (LS 6500, Beckman Coulter, Krefeld, Germany). The quantitative dpm values were normalized to the cellular protein content, obtained with the Pierce™ BCA Protein Assay Kit. The percentages of [^3^H]-labeled PLs in the media relative to the total radiolabeled PLs in the media and cell lysate were recorded.

### 2.11. Treatment of FLSs to Study PL Biosynthesis

Human FLSs from passage 3 or 4 were seeded into 6-well cell culture dishes at a density of 80,000 per well as described [10,32]. Briefly, cells were grown until confluence, analyzed via FACS, and then cultured for 24 h in 2 mL choline-depleted, phenol-red-free and serum-reduced DMEM (5% FBS), which was supplemented as described above, this time containing an additional 4 mg/L of folic acid. Furthermore, the media were changed, and the cells were labeled with 50 µg/mL [D9]-choline or [D4]-ethanolamine and treated simultaneously with 1 of the 5 lipid species, either 20 µM LPC 16:0, LPC 18:0, or LPC 18:1 or 10 µM LPA 16:0 or LPA 18:0 for 18 h. The FLSs were washed twice with PBS, lysed with 0.1% SDS, and sonicated, and the protein concentration was measured using the Pierce™ BCA Protein Assay Kit. The experiment was repeated 6 times using FLSs from 7 patients with knee OA (age: 71.7 ± 12.9 years, BMI: 29.4 ± 3.8 kg/m^2^, CRP: 3.98 ± 3.73 mg/L, 2 males and 5 females). Comorbidities included arterial hypertension (*n* = 5), hypothyroidism (*n* = 4), hyperlipidemia (*n* = 2), and fibromyalgia (*n* = 1).

### 2.12. MS Analysis of Stable Isotope-Labeled PLs

Lipids from stable isotope-labeled FLS lysates were extracted according to Bligh and Dyer [31] in the presence of non-naturally occurring internal lipid standards (Avanti Polar Lipids, Alabaster, AL, USA) and analyzed as described [10,32]. Briefly, PL species, unlabeled or labeled with stable isotopes, were analyzed by electrospray ionization tandem mass spectrometry (ESI-MS/MS; Quattro Ultima™ Triple Quadruple mass spectrometer, Micromass) [33]. A precursor ion mass scan of (*m*/*z*) 184 was used to detect PC, sphingomyelin (SM), and LPC, and a neutral loss scan of 141 was used to detect PE. Fragment ions of *m*/*z* 364, 380, and 382 were used to detect PE P-16:0, PE P-18:1, and PE P-18:0, respectively. A precursor ion scan of 193 and a neutral loss scan of 145 were applied to quantify [D9]-choline-labeled and [D4]-ethanolamine-labeled PLs.

The isotopic overlap of PL species was corrected, and a self-programmed Excel macro was used for data analysis [34]. A standard method for reporting PL species that are derived from MS was applied to annotate them [35]. The amount of cellular proteins was used to normalize quantitative values, expressed in nmol/mg protein. Only PL species that had concentrations above 1% of the corresponding PL class and were over 3-fold higher than the internal standard blank were considered.

### 2.13. Bioinformatics

We used Proteome Discoverer, version 2.5.0.400 (Thermo Fisher Scientific, Waltham, MA, USA) to access protein annotation from the web-based Gene Ontology (GO) database (http://geneontology.org/, accessed on 16 August 2022). To analyze the pathophysiological characterization of differentially regulated proteins, information on 3 annotations—biological processes, molecular functions, and cellular components—was obtained from the GO database. The GO Slim Terms (http://www.informatics.jax.org/gotools/MGI_GO_Slim.html, accessed on 16 August 2022) that were used for each annotation aspect originated from the Jackson Laboratory, which we modified with GO Slim Terms from the GO Enrichment Tool in Proteome Discovery according to protein enrichment and the implied relevance to OA.

### 2.14. Statistical Analysis

The statistical analysis and graphs were created using GraphPad Prism, version 9.5.0 for Windows (GraphPad Software, San Diego, CA, USA) and Proteome Discoverer, version 2.5.0.400 (Thermo Fisher Scientific, Waltham, MA, USA), respectively. The proteins for the proteomic analysis were derived from the cell extracts of treated or untreated FLS cultures, each derived from the same patient. The experiments were repeated 5 times; thus, a total of 6 biological replicates from cultured FLSs of 6 OA patients per treatment, each determined in duplicate, were analyzed (*n* = 6). Thus, proteomic data from 12 replicates per treated or untreated FLS culture were obtained.

To correct for skewness, the measured abundance ratios (ARs) were log-transformed prior to entering the variables in the analysis. The following prerequisites were applied to be further statistically analyzed: (1) The AR of the proteins was reproducibly regulated by LPC or LPA species, IL-1β, or IL-1β in the presence of LPC 16:0 above 1.2-fold or below 0.8-fold in at least 10 of the 12 replicates; (2) on average, the AR of proteins from FLSs that were treated with 1 of the LPC or LPA species had to be over 1.2-fold or below 0.8-fold; and (3) the average AR of proteins from FLSs that were treated with IL-1β with or without LPC 16:0 had to be elevated by at least 1.5-fold or decreased below 0.66-fold.

The adjusted *p*-values for the comparisons of the levels of treated versus untreated controls (Table S1) were obtained by means of repeated-measures one-way ANOVA, followed by Dunnett´s multiple comparisons test. The unadjusted *p*-values for the comparisons of 2 ARs (Table S2) or of the levels of proteins in FLSs that were treated with IL-1β alone versus those of the untreated control (Table S3) were obtained. For this purpose, multiple paired *t*-test analysis was performed using the 2-stage linear step-up procedure of Benjamini et al. [36] to control the false discovery rate (FDR), which was set to 10%. The ARs are presented as mean ± SD, and those in at least 10 of the 12 reproducibly regulated replicates are shown in bold (Tables S1–S3). The significance threshold was set to *p* ≤ 0.05.

The effects of LPC and LPA species on the biosynthesis and release of PLs were determined in cultured FLSs from the same patient. These experiments were repeated 5 or 6 times using cultured FLSs from 6 or 7 OA patients per treatment (*n* = 6–7). Repeated-measures one-way ANOVA, followed by Dunnett´s multiple comparisons test, was applied to log-transformed data to calculate whether LPCs and LPAs modulate the biosynthesis of PLs (Table 1) and the release of radiolabeled PLs from FLSs into nutrient media. The adjusted *p*-values are reported. In addition, Spearman correlation analysis was performed between the mean AR of select proteins and the mean fold-change in their corresponding mRNA level.

## 3. Results

### 3.1. MS Identification of Differentially and Reproducibly Regulated Proteins

By means of MS, 3960 master proteins were quantified in the extracts of FLSs after applying an FDR of less than 0.01, a minimum HT Sequest score of >30, and at least one identified unique peptide per protein. In addition, we eliminated haptoglobin and contaminants, according to the ThermoScientific database. The identified master proteins had to be upregulated by more than 1.2-fold or decreased by less than 0.8-fold in at least 10 of the 12 technical replicates of each treatment group to be considered reproducibly regulated.

**Figure 1:**
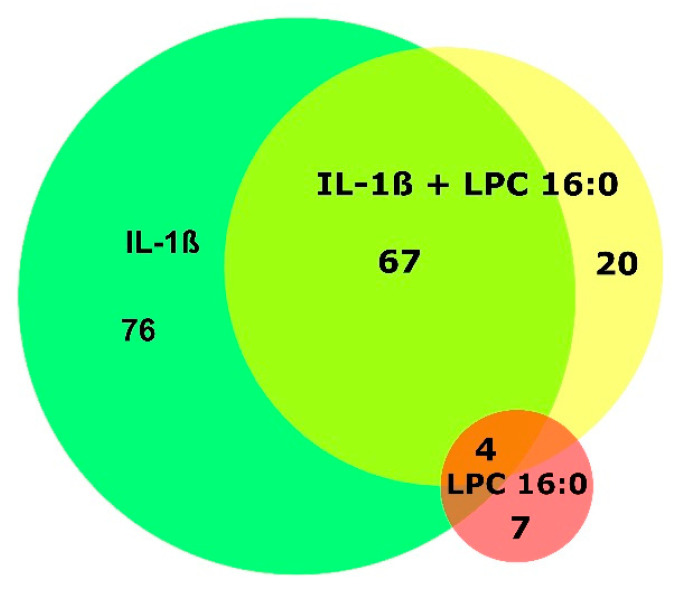
Venn diagram of 174 proteins reproducibly regulated by LPC 16:0 (red), IL-1β alone (green), and LPC 16:0 in the presence of IL-1β (yellow). The number of reproducibly regulated proteins is shown, whereas Tables S1–S3 display the AR of each protein with the results of the statistical analysis.

**Figure 2:**
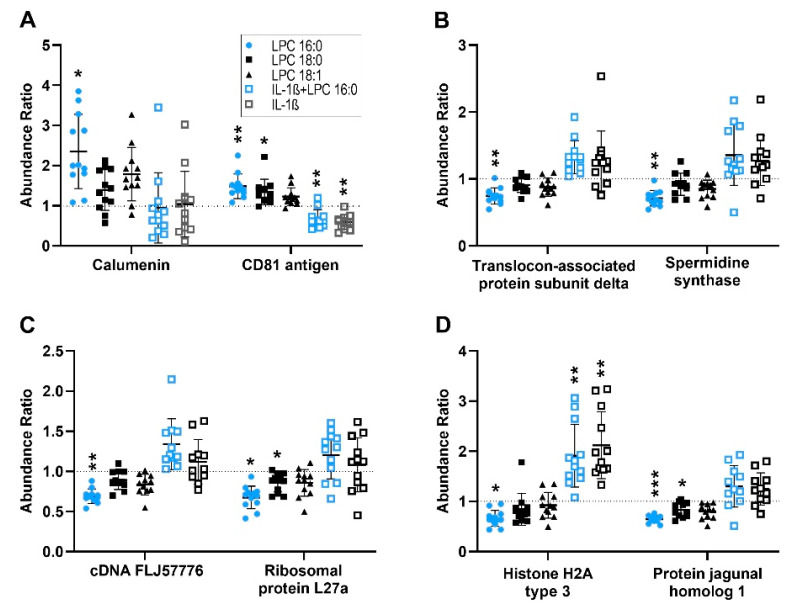
LPC 16:0 reproducibly and significantly regulates the level of 8 proteins in FLSs, namely (**A**) calumenin, CD81 antigen; (**B**) translocon-associated protein subunit delta, spermidine synthase; (**C**) cDNA FLJ57776, ribosomal protein L27a; (**D**) histone H2A type 3, and protein jagunal homolog 1. The effects of LPC 18:0, LPC 18:1, LPA 16:0, LPA 18:0, IL-1β, and LPC 16:0 in the presence of IL-1β are shown for comparison. Protein levels were determined by MS in 6 biological replicates, each being determined in duplicate. Data for biological and technical replicates are shown as dot plots and represent fold-change in protein abundances from treated FLSs versus that of their corresponding untreated controls (normalized to 1, indicated by a dotted line). Lines inside each figure denote the mean ± SD (*n* = 6). * 0.05 ≥ *p* > 0.01; ** 0.01 ≥ *p* >0.001; *** *p* ≤ 0.001.

LPC 16:0 reproducibly regulated eleven proteins, with no additional IL-1β added to the cells (Figure 1, Table S1). LPC 16:0 was the most active LPC species, significantly upregulating (A0A024R755, P60033) or downregulating (P51571, P19623, B4E2C1, Q9BQQ5, Q7L7L0, A0A3B3ITE9) eight proteins (Figure 2, Table S1). The levels of three proteins, although not reproducible, were significantly elevated (P60033) or decreased (Q9BQQ5, A0A3B3ITE9) by LPC 18:0. LPC 18:1 and the LPA species had no stimulatory or inhibitory effect on the level of any protein (Figure 2, Table S1). In the presence of IL-1β, LPC 16:0 did not have any additional effects. Thus, IL-1β appears to have a dominant effect, antagonizing even the stimulatory and inhibitory effects of LPC 16:0 on the expression of 11 proteins (Figure 2, Table S1).

LPC 16:0 was also tested in the presence of IL-1β, which has a central function in OA. Figure 1 shows the number of all proteins that were reproducibly regulated by LPC 16:0 alone and IL-1β with or without LPC 16:0. Overall, only LPC 16:0 reproducibly regulated 20 proteins in the presence of IL-1β, while IL-1β alone also affected them, though not reproducibly (Table S2). Comparing the ARs of both treatments, there were no significant differences in the expression of these proteins (Table S2). Conversely, 147 proteins were reproducibly regulated by IL-1β alone (Figure 1, Table S3), 71 of which were also reproducibly regulated by LPC 16:0 with IL-1β (Figure 1, Table S2). A comparison of the ARs of the proteins between FLSs that were treated with IL-1β alone versus LPC 16:0 plus IL-1β revealed no significant differences (Figure 1, Table S2). The levels of 119 of 147 proteins that were reproducibly regulated by IL-1β alone also rose significantly by at least 1.5-fold or decreased by 0.66-fold (Figure 1, Table S3).

### 3.2. Biological Functions of Differentially Regulated Proteins

**Figure 3:**
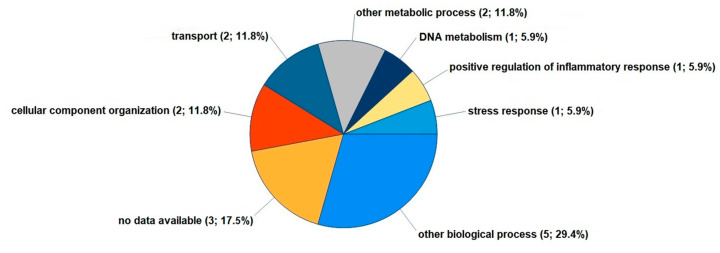
The biological processes of the 8 proteins significantly regulated in FLSs by LPC 16:0. Using the Gene Ontology (GO) database, Proteome Discoverer 2.5 provided the GO slim categories for proteins reproducibly upregulated at least 1.2-fold or downregulated 0.8-fold in FLSs treated with LPC 16:0 for 48 h. Further data on the 8 differentially expressed proteins are provided in Table S1.

According to the GO slim categories for the biological processes annotation, most of the eight proteins that were significantly regulated by LPC 16:0 are involved in the organization of the cellular component (Q7L7L0, A0A3B3ITE9), transport (P60033, B4E2C1), DNA metabolism (Q7L7L0), and the positive regulation of the inflammatory response (P60033) (Figure 3).

**Figure 4:**
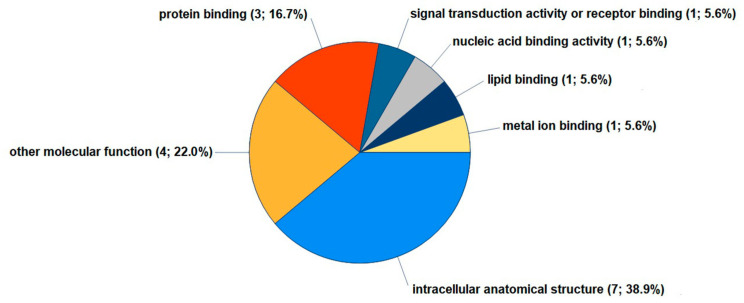
The molecular functions of the 8 proteins significantly regulated in FLSs by LPC 16:0. Using the GO database, Proteome Discoverer 2.5 provided the GO slim categories for proteins reproducibly upregulated at least 1.2-fold or downregulated 0.8-fold in FLSs treated with LPC 16:0 for 48 h. Further data on the 8 differentially expressed proteins are provided in Table S1.

For the molecular functions annotation, LPC 16:0 altered the expression of proteins that are involved in the intracellular anatomic structure (A0A024R755, P51571, P19623, B4E2C1, Q9BQQ5, Q7LL7L0, A0A3B3ITE9) and signal transduction or binding of receptors, proteins (P60033, P19623, Q7L7L0), nucleic acids (Q7L7L0), lipids (P60033), or metal ions (A0A024R755) (Figure 4).

**Figure 5:**
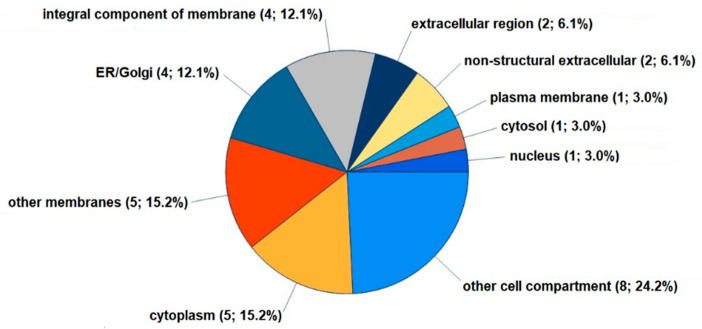
Localization of 8 proteins significantly regulated in FLSs by LPC 16:0 in cellular components. Using the GO database, Proteome Discoverer 2.5 provided the GO slim categories for proteins reproducibly upregulated at least 1.2-fold or downregulated 0.8-fold in FLSs treated with LPC 16:0 for 48 h. Further data on the 8 differentially expressed proteins are provided in Table S1.

The differentially expressed proteins resided in several cellular structures, many of which were integral components of a membrane (P60033, P51571, B4E2C1, A0A3B3ITE9), the plasma membrane (P60033), the ER/Golgi (A0A024R755, P51571, B4E2C1, A0A3B3ITE9), the nucleus (Q7L7L0), other cell compartments (all eight proteins), and the cytosol/cytoplasm (A0A024R755, P51571, P19623, B4E2C1, A0A3B3ITE9) (Figure 5).

However, IL-1β reproducibly and significantly modulated 119 proteins by at least 1.5-fold or less than 0.66-fold on average (Table S3). Due to the large number of proteins, the analysis of GO slim categories for the annotations biological processes (Figure S1), molecular functions (Figure S2), and cellular component (Figure S3) demonstrated a significant and diverse impact of IL-1β on the cultured FLSs.

With regard to biological processes, IL-1β significantly regulated at least 10 proteins per GO slim category, including stress response, the organization of cellular components, protein metabolism, signal transduction, transport, RNA metabolism, and transcription (Figure S1). Fewer than 10 proteins per GO slim category are involved in apoptosis, the response to reactive oxygen species, the positive regulation of inflammatory responses, or chronic inflammatory responses (Figure S1). For molecular functions, 91 proteins were involved in intracellular anatomical structure, and 46, 24, 17, and 7 proteins participated in the binding of proteins, metal ions, nucleic acids, and lipids, respectively (Figure S2). At least 20 proteins were related to cellular components, such as the cytoplasm/cytosol, membranes, nucleus/nucleoplasm, and ER/Golgi, and fewer than 15 proteins were related to the cytoskeleton, mitochondria, secretory vesicles, lysosomes, or lipid droplets (Figure S3).

### 3.3. RT-PCR Analysis of Differentially Regulated Proteins

To compare the levels of regulated proteins with their corresponding mRNA, 16 proteins that were reproducibly and/or significantly regulated by LPC 16:0 and/or IL-1β were quantified by reverse-transcription polymerase chain reaction (RT-PCR) (Tables S1–S3). The correlation analysis of a total of 56 comparisons between the mean AR and fold-change in mRNA expression resulted in a Spearman correlation coefficient of r = 0.55 (*p* < 0.001).

### 3.4. Biosynthesis and Release of PLs from FLS

Table 1 shows that only LPC 16:0 inhibited the biosynthesis of PC to 67 ± 19% (*p* = 0.009), SM to 59 ± 18% (*p* = 0.001), and LPC to 70 ± 14% (*p* = 0.001) compared to the untreated controls. Since LPC and SM are synthesized from PC, the ratios of the amounts of the newly synthesized PL classes to their newly synthesized precursor [D9]-PC were first calculated and are expressed in Table 1 as a percentage of [D9]-LPC or [D9]-SM from [D9]-PC. A statistical comparison with the untreated control revealed no significant differences, indicating that the incorporation of [D9]-choline into LPC and SM was not specifically inhibited. Rather, the reduced amounts of [D9]-LPC and [D9]-SM arose indirectly from the LPC-16:0-induced inhibition of the incorporation of [D9]-choline into [D9]-PC, which is the precursor of [D9]-LPC and [D9]-SM.

However, of the two LPC metabolites, only LPA 16:0 significantly inhibited the incorporation of [D9]-choline into [D9]-SM. This effect was small and appears to have resulted from a slightly inhibited [D9]-PC synthesis, even though this did not reach statistical significance (Table 1).

The content of lipid classes was determined in the cellular extract by MS after a thorough washing of the FLSs. The total LPC content of the treated FLSs increased significantly, indicating that the three externally added LPC species were taken up by the FLSs to different extents (Table 1).

**Figure 6:**
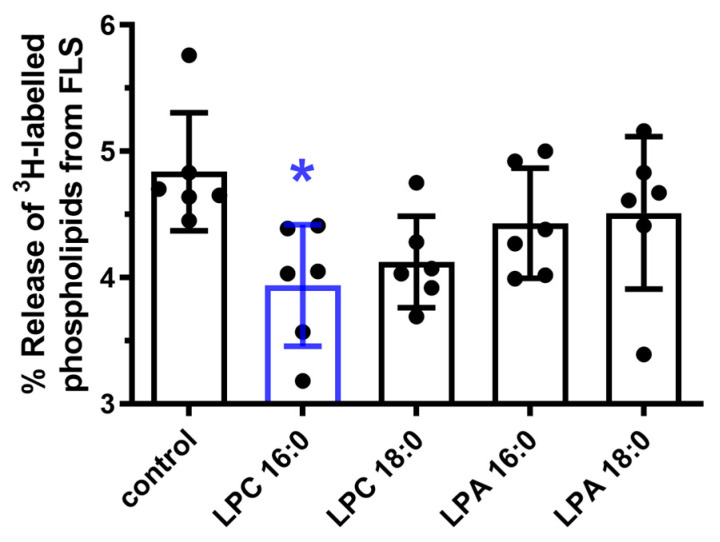
The release of radiolabeled PLs from human OA FLSs. Release of [^3^H]-labeled PLs into the media is expressed as percentage of PLs in media from total PLs (media + cell lysate) after normalization to total cellular protein content. Per well, FLSs were treated with 1 of the 4 lipid species, 20 µM of LPC species or 10 µM LPA species, or the vehicle as a control. The data are presented as dot plots, with lines denoting mean ± SD (*n* = 6). * *p* ≤ 0.05.

Figure 6 demonstrates that only LPC 16:0 significantly inhibited the release of [^3^H]-labeled PLs from prelabeled FLSs into the nutrient media, by approximately 19% (*p* = 0.012), indicating again that LPC 16:0 is the most bioactive LPC species tested.

## 4. Discussion

There is limited knowledge on the (patho)physiological impact of minor structural variations in lipids [37]. One of the most notable findings of our study is that even small chemical differences between individual LPC species have markedly different effects on protein expression in FLSs that are derived from human OA knee joints. Our study demonstrates that LPC 16:0 is the most active LPC species, in that it significantly and reproducibly regulated eight proteins, whereas LPC 18:0 significantly affected the expression of three proteins but not reproducibly; LPC 18:1 had no significant effect.

In various diseases, LPCs activate several downstream signaling pathways after binding to G-protein-coupled receptors and Toll-like receptors [21]. It is assumed that the pathophysiological function of LPC species is stored in their three-dimensional chemical structure [20,21,22,23,24]. Thus, the disparate effects of the three LPC species that we tested might be attributed to the differing affinities to their receptors in the respective signaling pathways and to their subcellular location.

Because the proinflammatory cytokine IL-1β has a central pathogenic function, especially during painful inflammatory episodes of OA, we also examined the effects of LPC 16:0 on the expression of proteins in the presence of IL-1β. Notably, we found that IL-1β antagonized the effects of LPC 16:0 on the expression of 11 proteins. The ARs of proteins from FLSs treated with LPC 16:0 and IL-1β were not different from those from FLSs treated with IL-1β alone, suggesting that this cytokine has a dominant effect on the expression of proteins in FLSs that LPC 16:0 can not modulate.

Furthermore, our proteomic study demonstrates the pleiotropic effects of IL-1β on overall protein expression in human FLSs and identifies proteins that are involved in diverse biochemical pathways. Such effects are not surprising and have been described for several articular cell types, such as chondrocytes, osteoblasts, osteoclasts, and synovial fibroblasts [38,39].

Only LPC 16:0 inhibited both the release of PLs from FLSs and the biosynthesis of PC, resulting in less SM and LPC being produced. None of the other LPC and LPA species tested exerted any effect on PLs. However, our MS analysis did not identify any significantly regulated proteins that are involved in the biosynthesis or release of PLs. Our data indicate that LPC 16:0 might have influenced the activity of enzymes and transporters that mediate the synthesis and release of PLs instead. Consistent with this model, LPA 16:0 and LPA 18:0 did not regulate any of the eight proteins that were reproducibly and significantly affected by their corresponding LPC species (16:0 and 18:0, respectively). Thus, neither LPA species contributed as LPC metabolites to the effects of their corresponding LPC species, as has been suggested for other cells [20]. Taken together, our data imply a negative feedback mechanism that likely prevents excessive levels of LPC species within OA joints. Future studies are required to show whether PLs are released directly or as part of lipid vesicles.

We have reported a lipidomic study [2] showing that the concentrations of all LPCs in human early and late OA SF are increased 2.8-fold and 4.8-fold, respectively, compared with those of healthy controls. For instance, we measured the median concentrations (nmol/mL) and interquartile ranges (IQR) for LPC 16:0, LPC 18:0, and LPC 18:1 of 24.7 (18.9–28.0), 10.8 (6.45–12.0), and 6.26 (4.94–8.78) in late OA SF, respectively [2]. Similarly, compared to human control SF, the levels of all LPA species rose by 2.7-fold in early OA SF and 4.0-fold in late OA SF [40]. The median concentrations (nmol/mL) and IQR for LPA 16:0 and LPA 18:0 were 1.37 (0.93–2.49) and 0.20 (0.13–0.33) in late OA SF, respectively [40]. Taken together, the LPC and LPA concentrations of 20 µM and 10 µM, respectively, added in the current proteomic study to the cultured FLS include the pathophysiological range reported for human OA SF.

By using 5% FBS in our culture media, these PLs were also introduced, but probably in much lower amounts than those added separately, although there seems to be no data available in the literature on FBS. Based on data from human serum or plasma, our serum-reduced culture medium contained the equivalent concentrations (nmol/mL) for LPC 16:0, LPC 18:0, LPC 18:1, LPA 16:0, and LPA 18:0 of approximately 3.6, 1.4, 0.9, 0.004, and 0.001 [41,42]. Whether the low PL concentrations added to the culture medium via 5% FBS already have an effect on FLS remains to be determined in further studies.

We identified eight proteins that were reproducibly and significantly upmodulated by at least 1.2-fold or downregulated by 0.8-fold by LPC 16:0. Our proteomic data were validated through the fact that we focused solely on these proteins, which were reproducibly regulated in our six biological replicates, each determined twice by MS. This approach was necessary because our preliminary experiments revealed that the MS data between biological and technical replicates have limited reproducibility. Thus, of the 12 replicates that were analyzed by MS per treatment, only the levels of proteins that were regulated in the same direction in at least 10 of the 12 replicates were considered reproducible and thus reliably upregulated or decreased.

Some significantly regulated proteins were further analyzed using quantitative RT-PCR. Compared with the 56 mean ARs of proteins in FLSs, the corresponding mRNA expression values correlated moderately (r = 0.55) with them. This is not unexpected, given that several studies have described a limited association between these two types of data [43,44], attributed to technical and biological causes, such as post-transcriptional regulatory mechanisms [43,44].

Our main aim was to identify significantly regulated proteins with potential pathophysiological relevance to OA. One of them is the cell surface antigen CD81 (P60033). The suppression of CD81 impeded the development of inflammatory arthritis in a rat model [45]. Further, through a DNA microarray analysis, CD81 was found to regulate the expression of such cytokines as IL-1β and TNFα in RA synoviocytes in vitro [45]. Notably, LPC 16:0 and LPC 18:0 stimulated the expression of this protein, whereas IL-1β downregulated CD81 significantly, which was not altered by the addition of LPC 16:0. Our data imply an autocrine mechanism of IL-1β in controlling its levels, although this hypothesis remains to be tested. Based on reports of lower concentrations of IL-1β in slightly more advanced stages of OA compared with early OA, our data suggest that both LPC species act as proinflammatory mediators only in later stages of OA, with a KL score above II [8,9].

In our study, the levels of calumenin were significantly elevated in cultured FLSs that were treated with LPC 16:0 and LPC 18:1, whereas IL-1β had no effect. Notably, using a proteomic approach, this protein was downregulated in the secretome of cultured osteoblasts that were derived from the sclerotic areas of OA subchondral bone compared with those of nonsclerotic regions [46]. Calumenin is a highly conserved calcium-binding protein in eukaryotes; however, its functions are largely unknown. Human calumenin is a member of the CREC family and is found throughout the human body, particularly in the endoplasmic reticulum, where it is involved in protein folding and sorting [47]. Further, calumenin inhibits extracellular signal-regulating kinase 1 and 2 (ERK1/2) phosphorylation and cell migration by protecting fibulin-1 from cleavage by MMP-13 [48]. Whether both LPC species inhibit ERK1/2 through calumenin in human FLSs requires further study.

LPC 16:0 but not IL-1β decreased the level of spermidine synthase. This enzyme (EC 2.5.1.16) localizes to the cytosol and nucleoplasm and catalyzes the transfer of a propylamine group from S-adenosylmethionine to putrescine during the synthesis of the polyamine spermidine [49]. The polyamine metabolism is tightly regulated to maintain its homeostasis and adapt its levels to the physiological requirements of cells [50,51]. In this context, it is interesting to note a study with transgenic mice that showed that despite a marked increased spermidine synthase levels, its activity was unaffected, and even a slightly decreased spermidine level was measured [52]. However, spermidine was not detected in our study.

The expression of B4E2C1, a protein that is similar to transmembrane emp24 domain-containing protein 7 (TMED7), was slightly inhibited by LPC 16:0, whereas IL-1β had no effects. TMED7 is found predominantly in the Golgi apparatus and ER, where it mediates the transport of such proteins as Toll-like receptor 4 to the cell surface [53]. Notably, TMED7 has been reported to be a cis-related gene of NF-κB [54]. Studies in alveolar type-II cells have shown that microRNA-340-5p has anti-inflammatory effects by inhibiting TMED7 and thus the activation of NF-κB [55]. Whether LPC 16:0 also modulates the TMED7/NF-κB axis in human FLSs remains to be determined.

LPC 16:0 inhibited the expression of the ribosomal protein L27a, translocon-associated protein, histone H2A type 3, and the protein jagunal homolog 1 (JAGN1), whereas only histone H2A type 3 was stimulated by IL-1β. L27a resides in the cytoplasm in most cells and is involved in translation. However, there are limited data on this ribosomal protein, as is the case for translocon-associated protein and JAGN1. Translocon-associated protein is located in the ER, where it binds calcium to the ER membrane to regulate the retention of resident proteins [56]. This protein is involved in congenital disorder of glycosylation 1Y (SSR4-CDG; *CDG*-Iy) [57]. JAGN1 is a ubiquitously expressed transmembrane protein that functions in early secretory pathways and is required for neutrophil differentiation and survival. A recent study on B cells found that JAGN1 regulates humoral immunity in mice and humans [58]. Histone H2A packs DNA into nucleosomes and is thus responsible for their shape and structure. Histone H2A regulates transcription, DNA synthesis, and chromosome stabilization.

In conclusion, our in vitro study was motivated by the limited data on the function of individual LPC species in articular joints. Using a novel proteomic MS analysis, we detected for the first time the distinct protein expression patterns of FLSs induced by three LPC species, of which LPC 16:0 is the most active. Our MS approach generated a large dataset, but only eight proteins were found to be significantly and reproducibly regulated by LPC 16:0. Although none have been described in joints or OA, CD81, calumenin, and TMED7 are promising candidates for further studies, focusing in particular on their potential ability to influence inflammation and degradation processes, to better understand their role in joint disease. Our data suggest that, despite differing only slightly chemically, individual LPC species can differ tremendously in terms of their activity in articular joint cells, especially at low levels of IL-1β, as seen in the more advanced stages of OA.

## Figures and Tables

**Table 1:** Effect of lipid species on the level of newly synthesized PL classes.

Treatment	[D9]-PC (nmol/mg) PC Total (nmol/mg)	[D9]-SM (nmol/mg) SM Total (nmol/mg) *[D9]-SM/* *[D9]-PC* (%)	[D9]-LPC (nmol/mg) LPC Total (nmol/mg) *[D9]-LPC/* *[D9]-PC* (%)	[D4]-PE (nmol/mg) PE Total (nmol/mg)	[D4]-PE P (nmol/mg) PE P Total (nmol/mg)
**Control #1**	**5.37 ± 1.34**	**0.31 ± 0.07**	**0.05 ± 0.01**	**4.32 ± 0.63**	**3.29 ± 0.72**
	75.1 ± 5.94	32.9 ± 3.72	1.26 ± 0.19	17.9 ± 1.67	22.9 ± 2.02
		*5.73* ± *0.33*	*0.87* ± *0.10*		
LPC 16:0	3.69 ± 1.58 **	0.19 ± 0.08 **	0.03 ± 0.01 **	**5.78 ± 1.55**	**3.91 ± 1.09**
	81.0 ± 19.0	30.3 ± 9.08	5.77 ± 1.22 ***	19.4 ± 5.37	22.7 ± 5.77
		*5.05* ± *0.76*	*0.94* ± *0.17*		
LPC 18:0	**4.60 ± 1.59**	**0.23 ± 0.10**	**0.04 ± 0.01**	**5.32 ± 2.60**	**3.80 ± 2.04**
	89.8 ± 25.6	35.3 ± 10.3	5.73 ± 1.89 **	21.4 ± 4.51	26.3 ± 7.07
		*4.94* ± *0.74*	*0.89* ± *0.08*		
LPC 18:1	**5.51 ± 2.00**	**0.31 ± 0.11**	**0.05 ± 0.02**	**4.45 ± 2.12**	**3.27 ± 1.48**
	76.1 ± 22.1	31.7 ± 11.4	3.65 ± 1.93 *	17.1 ± 4.97	21.9 ± 6.48
		*5.78* ± *0.85*	*0.86* ± *0.09*		
**Control #2**	**7.15 ± 1.71**	**0.43 ± 0.11**	**0.06 ± 0.01**	**5.61 ± 0.65**	**4.19 ± 0.61**
	84.5 ± 6.35	36.8 ± 6.74	1.35 ± 0.29	19.6 ± 3.36	25.2 ± 3.04
		*6.05* ± *0.54*	*0.85 ± 0.10*		
LPA 16:0	**6.24 ± 1.70**	0.35 ± 0.12 *	**0.06 ± 0.01**	**5.66 ± 0.69**	**4.32 ± 0.62**
	78.8 ± 7.1	34.0 ± 4.62	1.40 ± 0.29	18.9 ± 2.15	24.0 ± 2.28
		*5.67* ± *0.88*	*0.90* ± *0.07*		
LPA 18:0	**7.15 ± 1.49**	**0.43 ± 0.08**	**0.07 ± 0.01**	**6.22 ± 0.82**	**4.70 ± 0.84**
	80.4 ± 10.3	35.4 ± 8.47	1.48 ± 0.38	18.8 ± 3.66	24.1 ± 4.02
		*6.10* ± *0.47*	*0.92* ± *0.11*		

The quantitative values obtained from stable isotope-labelled PL classes were normalized with respect to the cellular protein content (nmol/mg protein) and are indicated in bold. The total amount of labelled and unlabeled PL classes found in cellular lysates were normalized to the cellular protein content and are expressed as nmol/mg protein. The percentages of [D9]-SM (nmol/mg) to [D9]-PC (nmol/mg) and [D9]-LPC (nmol/mg) to [D9]-PC (nmol/mg) were calculated and are indicated in italics. Data are presented as means ± SDs (*n* = 7). Significantly different compared to untreated control: * 0.05 > *p* > 0.01; ** 0.001 ≤ *p* ≤ 0.01; *** *p* < 0.001. Control #1—untreated control containing only the vehicle of the added LPC species; control #2—untreated control containing only the vehicle of the added LPA species; PC = phosphatidylcholine; PE = phosphatidylethanolamine; PE P = phosphatidylethanolamine-based plasmalogens; SM = sphingomyelin; LPC = lysophosphatidylcholine.

## Data Availability

The data in this study are available on reasonable request from the corresponding author.

## Supplementary Materials

The following supporting information can be downloaded at: https://www.mdpi.com/article/10.3390/cells12131743/s1, Figure S1: Biological processes of 119 proteins significantly regulated in FLSs by IL-1β; Figure S2: Molecular functions of 119 proteins significantly regulated in FLSs by IL-1β; Figure S3: Localization in cellular components of 119 proteins significantly regulated in FLSs by IL-1β; Table S1: Proteins reproducibly regulated by LPC 16:0 in human FLSs; Table S2: The 91 proteins reproducibly regulated by LPC 16:0 in the presence of IL-1β in human FLSs; Table S3: The 147 proteins reproducibly regulated by IL-1β alone in human FLSs.
