# Supplementary material for: Functional Characterization of Lysophospholipids by Proteomic and Lipidomic Analysis of Fibroblast-like Synoviocytes
Source: Cells. 2023 Jun 29;12(13):1743. doi: 10.3390/cells12131743 (PMC10340184; doi:10.3390/cells12131743)
Supplement: Supplementary file 1 [file cells-12-01743-s001.zip › cells-2456618-supplementary.pdf]

Supplementary Information

Functional Characterization of Lysophospholipids by Proteomic and Lipidomic Analysis of Fibroblast-Like Synoviocytes

Thomas Timm [1], Christiane Hild [2], Gerhard Liebisch [3], Markus Rickert [2], Guenter Lochnit [1] and Juergen Steinmeyer [2,*]

[1] Protein Analytics Group, Institute of Biochemistry, Justus Liebig University Giessen, 35392 Giessen, Germany

[2] Laboratory for Experimental Orthopedics, Department of Orthopedics, Justus Liebig University Giessen, 35392 Giessen, Germany

[3] Department for Clinical Chemistry and Laboratory Medicine, University Hospital Regensburg, 93053 Regensburg, Germany

* Correspondence: juergen.steinmeyer@ortho.med.uni-giessen.de

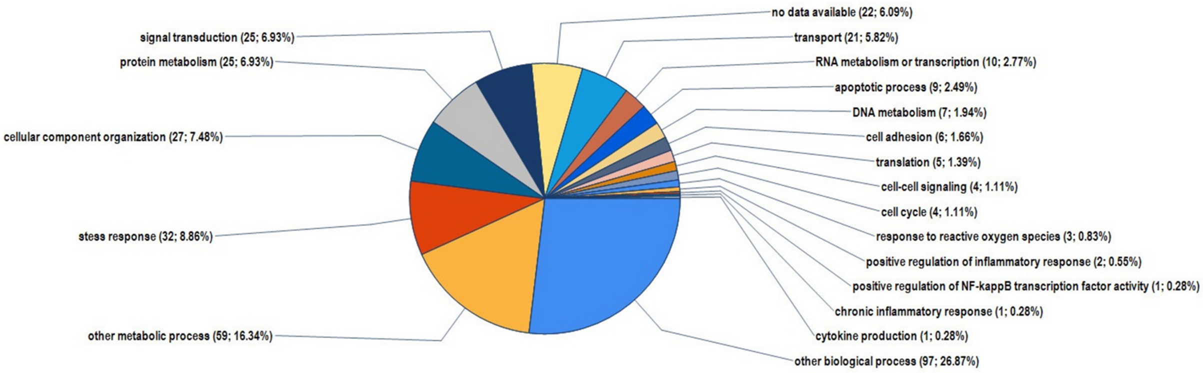

Figure S1. Biological processes of 119 proteins significantly regulated in FLSs by IL-1β. Using the GO database, Proteome Discoverer 2.5 provided the GO slim categories for proteins reproducibly upregulated at least 1.5-fold or downregulated 0.66-fold in FLSs treated with IL-1β for 48 h. Further data on the differentially expressed proteins are provided in Table S3.

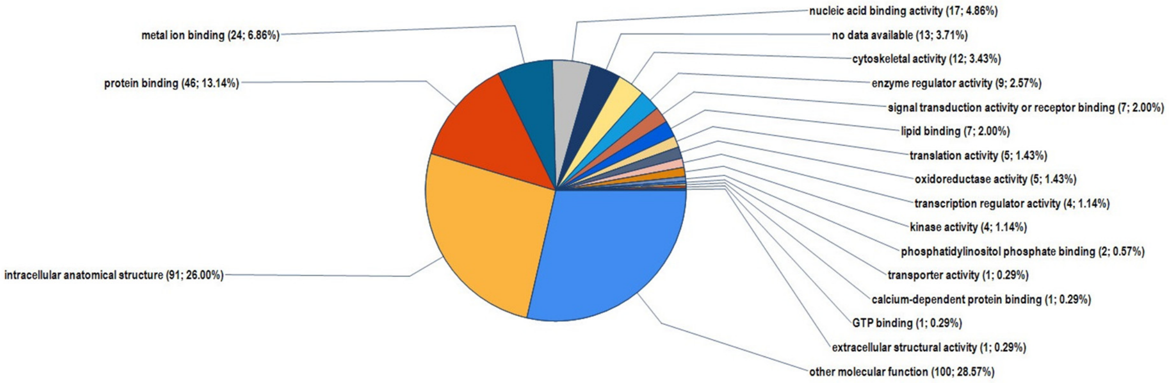

Figure S2. Molecular functions of 119 proteins significantly regulated in FLSs by IL-1β. Using the GO database, Proteome Discoverer 2.5 provided the GO slim categories for proteins being reproducibly upregulated at least 1.5-fold or downregulated 0.66-fold in FLSs treated with IL-1β for 48 h. Further data on the differentially expressed proteins are provided in Table S3.

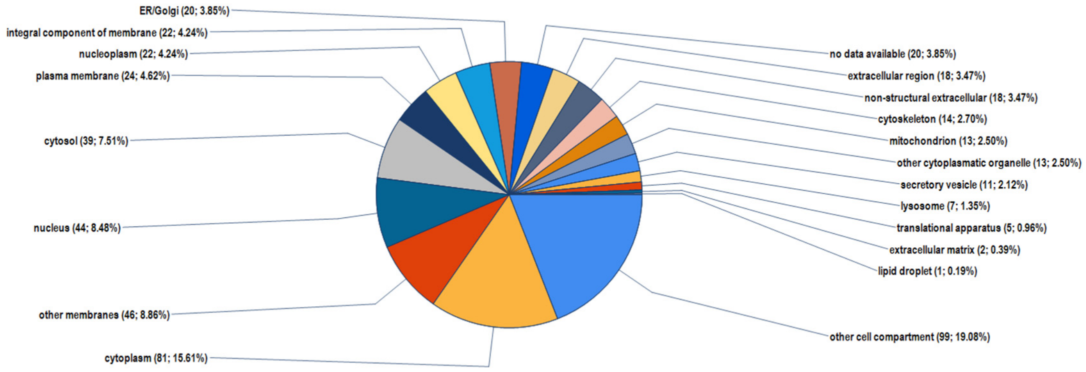

Figure S3. Localization in cellular components of 119 proteins significantly regulated in FLSs by IL-1β. Using the GO database, Proteome Discoverer 2.5 provided the GO slim categories for proteins upregulated at least 1.5-fold or downregulated 0.66-fold in FLSs treated with IL-1β for 48 h. Further data on the differentially expressed proteins are provided in Table S3.

Table S1. Proteins reproducibly regulated by LPC 16:0 in human FLSs.

Accession ID	Gene	Protein name	AR<br>LPC 16:0	AR<br>LPC 18:0	AR<br>LPC 18:1	AR<br>LPA 16:0	AR<br>LPA 18:0	AR<br>IL-1β +<br>LPC 16:0	AR<br>IL-1β
**A0A024R755**	*CALU*	Calumenin	**2.35 ± 0.93**<br>*<br>*(0.89±0.18)*	1.39 ± 0.51<br>*(0.87±0.18)*	**1.79 ± 0.67**<br>*(0.85±0.17)*	1.02 ± 1.16	1.39 ± 0.96	0.95 ± 0.88<br>*(0.81±0.18)*	1.04 ± 0.82<br>*(0.83±0.18)*
**Q8NI22**	*MCFD2*	Multiple coagulation factor deficiency protein 2	**1.67 ± 0.58**<br>*(0.82±0.13)*	1.26 ± 0.58<br>*(0.79±0.17)*	1.30 ± 0.27<br>*(0.83±0.22)*	1.27 ± 1.45	1.58 ± 1.40	**0.47 ± 0.52**<br>*(0.65±0.20)**	**0.39 ± 0.18**<br>**<br>*(0.72±0.20)*
**P07951-3**	*TPM2*	Isoform 3 of Tropomyosin beta chain	**1.55 ± 0.40**<br>*(0.80±0.12)*	1.24 ± 0.70<br>*(0.84±0.09)**	1.11 ± 0.19<br>*(1.02±0.17)*	1.18 ± 0.70	1.17 ± 0.52	**0.36 ± 0.31**<br>*<br>*(0.43±0.13)*<br>**	**0.21 ± 0.06**<br>***<br>*(0.43±0.11)*<br>**
**P60033**	*CD81*	CD81 antigen	**1.48 ± 0.31**<br>**<br>*(0.80±0.21)*	1.34 ± 0.32<br>*<br>*(0.83±0.18)*<br>*	1.23 ± 0.22<br>*(0.72±0.15)*<br>*	1.11 ± 0.56	1.25 ± 0.38	0.67 ± 0.23<br>**<br>*(0.33±0.09)*<br>**	0.59 ± 0.18<br>**<br>*(0.54±0.15)*<br>**
**Q86YQ0**	*HZGJ*	HZGJ	**1.41 ± 0.34**	1.12 ± 0.33	1.13 ± 0.23	0.98 ± 0.40	1.12 ± 0.38	**0.41 ± 0.49**<br>*	**0.22 ± 0.13**<br>***
**P51571**	*SSR4*	Translocon-associated protein subunit delta	**0.75 ± 0.12**<br>**<br>*(0.81±0.17)*	0.91 ± 0.11<br>*(0.80±0.13)*<br>**	0.88 ± 0.13<br>*(0.79±0.06)*<br>**	1.13 ± 0.38	0.92 ± 0.21	1.33 ± 0.25<br>*(0.89±0.18)*	1.27 ± 0.45<br>*(0.90±0.19)*
**P19623**	*SRM*	Spermidine synthase	**0.71 ± 0.12**<br>**<br>*(0.76±0.16)*	0.92 ± 0.17<br>*(0.90±0.27)*	0.85 ± 0.13<br>*(0.88±0.22)*	1.16 ± 0.29	0.98 ± 0.25	1.36 ± 0.45<br>*(0.82±0.45)*	1.28 ± 0.37<br>*(0.76±0.34)*

**B4E2C1**		cDNA FLJ57776, highly similar to Transmembrane emp24 domain-containing protein 7	**0.69 ± 0.09**<br>**<br>*(0.78±0.22)*	0.89 ± 0.12<br>*(0.78±0.09)*<br>***	0.84 ± 0.13<br>*(0.76±0.15)*	1.30 ± 0.60	0.90 ± 0.25	1.34 ± 0.32<br>*(0.82±0.16)*	1.12 ± 0.28<br>*(0.99±0.18)*
**Q9BQQ5**	*L27a*	Ribosomal protein L27a	**0.67 ± 0.14**<br>*<br>*(0.67±0.25)*	0.86 ± 0.12<br>*<br>*(0.75±0.29)*	0.86 ± 0.17<br>*(0.86±0.21)*	1.05 ± 0.23	0.95 ± 0.23	1.20 ± 0.30<br>*(0.50±0.12)*<br>***	1.08 ± 0.34<br>*(0.54±0.12)*<br>**
**Q7L7L0**	HIST3H2A	Histone H2A type 3	**0.67 ± 0.16**<br>*<br>(0.88±0.20)	0.84 ± 0.32<br>(0.83±0.24)	0.93 ± 0.26<br>(0.97±0.30)	1.01 ± 0.42	1.00 ± 0.47	**1.91 ± 0.63**<br>**<br>(0.64±0.29)	**2.12 ± 0.67**<br>**<br>(0.75±0.42)
**A0A3B3ITE9**	*JAGN1*	Protein jagunal homolog 1	**0.65 ± 0.07**<br>***<br>*(0.67±0.18)*<br>*	0.83 ± 0.15<br>*<br>*(0.77±0.15)*	0.80 ± 0.15<br>*(0.86±0.19)*	1.23 ± 0.58	0.89 ± 0.22	1.30 ± 0.41<br>*(0.85±0.29)*	1.25 ± 0.32<br>*(0.99±0.31)*

Proteins are ordered according to their mean abundance ratio (AR) in LPC 16:0 treated FLSs. The ARs of protein levels in FLSs treated with LPCs, LPAs, and/or IL-1β are shown relative to the abundance of their untreated controls. Accession IDs, gene symbols, and protein names were obtained from UniProt Knowledgebase. The ARs are presented as mean ± SD, and those reproducibly regulated in at least 10 of 12 replicates are shown in bold. The mRNA expression of select proteins was determined by RT-PCR using the $2^{-\Delta\Delta Ct}$ method. The data are presented in brackets and cursive style as mean ± SD of the fold-change in mRNA expression relative to control (normalized to 1). *0.05 ≥ P > 0.01; **0.01 ≥ P >0.001; ***P ≤ 0.001.

Table S2. The 91 proteins reproducibly regulated by LPC 16:0 in the presence of IL-1β in human FLSs.

**Accession ID**	**Gene**	**Protein name**	**AR IL-1β**	**AR IL-1β + LPC 16:0**
**A0A024R718**	*PBEF1*	Nicotinamide phosphoribosyltransferase	**5.78 ± 2.60**	**5.11 ± 2.86**
**O43570**	*CA12*	Carbonic anhydrase 12	**4.16 ± 2.00**	**4.09 ± 2.09**
**A0A024R969**	*CHI3L1*	Chitinase-3-like protein 1	**3.76 ± 4.47**	**4.03 ± 6.09**
**O60488**	*ACSL4*	Long-chain-fatty-acid--CoA ligase 4	**5.06 ± 2.15**	**3.90 ± 2.55**
**Q03169**	*TNFAIP2*	Tumor necrosis factor alpha-induced protein 2	**3.20 ± 1.46**	**3.40 ± 1.68**
**Q6FI13**	*H2AC18; H2AC19*	Histone H2A type 2-A	**4.58 ± 2.10**	**3.30 ± 1.33**
**B1AP13**	*CD55*	Complement decay-accelerating factor	**3.47 ± 2.68**	**3.25 ± 2.79**
**P47712**	*PLA2G4A*	Cytosolic phospholipase A2	**3.41 ± 1.54**	**3.25 ± 1.73**
**P17676**	*CEBPB*	CCAAT/enhancer-binding protein beta	**2.85 ± 0.99**	**3.15 ± 1.18**
**Q7Z7M4**	*SOD2*	Superoxide dismutase (Fragment)	**3.36 ± 1.35**	**3.01 ± 1.28**
**O95379**	*TNFAIP8*	Tumor necrosis factor alpha-induced protein 8	**2.81 ± 1.13**	**2.93 ± 1.19**
**E5KRG5**	*TYMP*	Thymidine phosphorylase	**2.72 ± 0.44**	**2.84 ± 0.79**
**A0A0G2JH37**	*TAPBP*	Tapasin	**2.26 ± 0.84**	**2.65 ± 1.00**
**O00469-2**	*PLOD2*	Isoform 2 of Procollagen-lysine,2-oxoglutarate 5-dioxygenase 2	**2.67 ± 1.10**	**2.52 ± 1.06**
**A0A024R8D5**		HCG30600, isoform CRA_a	**2.53 ± 1.35**	**2.35 ± 1.83**
**A0A0S2Z4X9**	*GFPT2*	Glutamine--fructose-6-phosphate transaminase (isomerizing) (Fragment)	**2.47 ± 1.11**	**2.20 ± 1.08**
**Q8IWB1**	*ITPRIP*	Inositol 1,4,5-trisphosphate receptor-interacting protein	**2.39 ± 1.09**	**2.16 ± 0.94**
**A0A024QZM9**	*PLAU*	Urokinase-type plasminogen activator	**1.98 ± 0.54**	**2.16 ± 0.54**
**F5GZS6**	*SLC3A2*	4F2 cell-surface antigen heavy chain	**2.42 ± 0.80**	**2.13 ± 0.86**
**Q04828**	*AKR1C1*	Aldo-keto reductase family 1 member C1	**2.17 ± 1.00**	**2.05 ± 0.81**
**Q8WUJ3**	*CEMIP*	Cell migration-inducing and hyaluronan-binding protein	**2.10 ± 0.62**	**2.02 ± 0.53**
**A0A346HG00**	*HLA-A*	MHC class I antigen	**2.09 ± 0.67**	**1.92 ± 0.90**
**Q7L7L0**	*H2AW*	Histone H2A type 3	**2.12 ± 0.67**<br>*(0.75±0.42)*	**1.91 ± 0.63**<br>*(0.64±0.29)*
**Q5H9B4**	*TIMP1*	Metalloproteinase inhibitor 1 (Fragment)	**1.72 ± 0.39**	**2.06 ± 1.06**
**P02671**	*FGA*	Fibrinogen alpha chain	**1.73 ± 0.67**	**2.04 ± 0.89**
**A0A024RAV0**	*BCAT1*	Branched-chain-amino-acid aminotransferase	**1.83 ± 0.42**	**1.98 ± 0.56**
**A0A024R1M8**	*APOL2*	Apolipoprotein L, 2, isoform CRA_a	**1.84 ± 0.29**	**1.83 ± 0.44**
**P20645**	*M6PR*	Cation-dependent mannose-6-phosphate receptor	**1.68 ± 0.36**	**1.77 ± 0.32**
**A0A024R6R4**	*MMP2*	72 kDa gelatinase	**1.88 ± 0.48**	**1.76 ± 0.75**
**P19838**	*NFKB1*	Nuclear factor NF-kappa-B p105 subunit	**1.65 ± 0.31**	**1.75 ± 0.47**
**Q14195-2**	*DPYSL3*	Isoform LCRMP-4 of Dihydro-pyrimidinase-related protein 3	**1.78 ± 0.41**	**1.74 ± 0.45**
**A0A024QZ89**	*COG4*	Component of oligomeric Golgi complex 4	1.47 ± 0.47	**1.68 ± 0.55**

**P09486**	*SPARC*	SPARC	1.48 ± 0.71	**1.64 ± 0.59**
**P13674**	*P4HA1*	Prolyl 4-hydroxylase subunit α-1	1.49 ± 0.36	**1.61 ± 0.68**
**B2R4R9**	*RPS28*	40S ribosomal protein S28	**1.58 ± 0.43**	**1.60 ± 0.44**
**P11498**	*PC*	Pyruvate carboxylase, mitochondrial	**1.64 ± 0.33**	**1.59 ± 0.62**
**H7BZJ3**	*PDIA3*	Protein disulfide-isomerase A3 (Fragment)	1.48 ± 0.11	**1.58 ± 0.13**
**A0A2R8Y855**	*SMARCE1*	SWI/SNF-related matrix-associated actin-dependent regulator of chromatin subfamily E member 1	1.33 ± 0.28	**1.56 ± 0.55**
**O14579**	*COPE*	Coatomer subunit epsilon	1.46 ± 0.63	**1.53 ± 0.43**
**Q13217**	*DNAJC3*	DnaJ homolog subfamily C member 3	**1.71 ± 0.50**	**1.52 ± 0.98**
**Q8WUW1**	*COG4*	Protein BRICK1	1.29 ± 0.36	**1.52 ± 0.41**
**Q2TNB3**		Cell migration-inducing protein 22	1.45 ± 0.21	**1.52 ± 0.28**
**Q15819**	*UBE2V2*	Ubiquitin-conjugating enzyme E2 variant 2	0.68 ± 0.14	**0.66 ± 0.18**
**Q9UHQ9**	*CYB5R1*	NADH-cytochrome b5 reductase 1	**0.66 ± 0.16**	**0.66 ± 0.28**
**Q9NVD7**	*PARVA*	Alpha-parvin	**0.66 ± 0.11**	**0.66 ± 0.19**
**B2RTQ2**	*TBC1D2B*	TBC1 domain family, member 2B, isoform CRA_b	**0.56 ± 0.24**	**0.66 ± 0.37**
**J3QL71**	*SCRN2*	Secernin-2	0.75 ± 0.37	**0.65 ± 0.62**
**Q9BVJ8**	*HEXA*	Beta-N-acetylhexosaminidase (Fragment)	**0.62 ± 0.10**	**0.65 ± 0.12**
**Q9Y376**	*CAB39*	Calcium-binding protein 39 1	**0.64 ± 0.15**	**0.64 ± 0.23**
**P29966**	*MARCKS*	Myristoylated alanine-rich C-kinase substrate	0.73 ± 0.20	**0.63 ± 0.27**
**O95373**	*IPO7*	Importin-7	**0.62 ± 0.11**	**0.63 ± 0.25**
**B5MC59**	*RPA3*	Replication protein A 14 kDa subunit	0.67 ± 0.17	**0.62 ± 0.26**
**D3DUZ3**	*IFI16*	Interferon, gamma-inducible protein 16, isoform CRA_a	**0.62 ± 0.20**	**0.62 ± 0.29**
**A0A384MTY2**		Epididymis secretory sperm binding protein	**0.60 ± 0.06**	**0.62 ± 0.11**
**Q16531**	*DDB1*	DNA damage-binding protein 1	**0.59 ± 0.07**	**0.62 ± 0.17**
**O75821**	*EIF3G*	Eukaryotic translation initiation factor 3 subunit G	**0.56 ± 0.14**	**0.61 ± 0.31**
**B4DKX4**		Programmed cell death protein 4	**0.55 ± 0.11**	**0.61 ± 0.26**
**V9HW48**	*HEL-S-115*	SH3 domain-binding glutamic acid-rich-like protein	**0.55 ± 0.16**	**0.61 ± 0.25**
**Q6NS36**	*FTH1*	Ferritin (Fragment)	**0.47 ± 0.08**	**0.61 ± 0.26**
**A0A024R3P9**	*ACBD3*	Acyl-Coenzyme A binding domain containing 3, isoform CRA_a	**0.60 ± 0.18**	**0.59 ± 0.27**
**Q13617**	*CUL2*	Cullin-2	**0.67 ± 0.17**	**0.58 ± 0.29**
**A0A024R125**	*PRKAG1*	5'-AMP-activated protein kinase subunit gamma-1	0.69 ± 0.21	**0.58 ± 0.32**
**Q6NUK1**	*SLC25A24*	Calcium-binding mitochondrial carrier protein SCaMC-1	0.68 ± 0.18	**0.58 ± 0.23**
**A0A024R1A4**	*UBE2L3*	Ubiquitin-conjugating enzyme E2L 3, isoform CRA_a	**0.52 ± 0.13**	**0.57 ± 0.16**
**B8ZWD1**	*DBI*	Acyl-CoA-binding protein	**0.44 ± 0.14**	**0.57 ± 0.31**
**Q6GMV3**	*PTRHD1*	Putative peptidyl-tRNA hydrolase PTRHD1	**0.57 ± 0.15**	**0.56 ± 0.24**
**Q13232**	*NME3*	Nucleoside diphosphate kinase 3	0.62 ± 0.17	**0.56 ± 0.23**
**Q9BVG4**	*PBDC1*	Protein PBDC1	0.75 ± 0.12	**0.54 ± 0.27**

**A8K651**		Complement component 1 Q subcomponent-binding protein, mitochondrial	**0.40 ± 0.15**	**0.54 ± 0.36**
**Q59G24**		Activated RNA polymerase II transcriptional coactivator p15 (Fragment)	**0.62 ± 0.17**	**0.53 ± 0.24**
**Q7Z2W9**	*MRPL21*	39S ribosomal protein L21, mitochondrial	0.65 ± 0.13	**0.52 ± 0.28**
**A0A1B0GVH5**	*FTO*	Alpha-ketoglutarate-dependent dioxygenase FTO	**0.53 ± 0.20**	**0.51 ± 0.33**
**P17900**	*GM2A*	Ganglioside GM2 activator	**0.50 ± 0.10**	**0.51 ± 0.18**
**Q53FX5**		Lin-7 homolog C variant (Fragment)	**0.56 ± 0.29**	**0.50 ± 0.29**
**P09012**	*SNRPA*	U1 small nuclear ribonucleoprotein A	0.61 ± 0.18	**0.49 ± 0.21**
**Q7L5D6**	*GET4*	Golgi to ER traffic protein 4 homolog	**0.42 ± 0.29**	**0.47 ± 0.65**
**Q8NI22**	*MCFD2*	Multiple coagulation factor deficiency protein 2	**0.39 ± 0.18** *(0.72±0.20)*	**0.47 ± 0.52** *(0.65±0.20)*
**P0DP25**	*CALM3*	Calmodulin-3	**0.37 ± 0.11**	**0.43 ± 0.21**
**P54727**	*RAD23B*	UV excision repair protein RAD23 homolog B	**0.46 ± 0.12**	**0.42 ± 0.13**
**Q86YQ0**	*HZGJ*	HZGJ	**0.22 ± 0.13**	**0.41 ± 0.49**
**B4DTX4**		cDNA FLJ59756, highly similar to Ras GTPase-activating protein 1	**0.35 ± 0.22**	**0.41 ± 0.24**
**P05114**	*HMGN1*	Non-histone chromosomal protein HMG-14	**0.50 ± 0.16**	**0.40 ± 0.20**
**P54725**	*RAD23A*	UV excision repair protein RAD23 homolog A	**0.41 ± 0.27**	**0.38 ± 0.26**
**Q9Y2Z0**	*SUGT1*	Protein SGT1 homolog	0.53 ± 0.36	**0.38 ± 0.29**
**Q6IPW4**	*NDUFV2*	NADH dehydrogenase [ubiquinone] flavoprotein 2, mitochondrial	**0.39 ± 0.32**	**0.37 ± 0.31**
**P07951-3**	*TPM2*	Isoform 3 of Tropomyosin ß chain	**0.21 ± 0.06**	**0.36 ± 0.31**
**P84157-3**	*MXRA7*	Isoform 3 of Matrix-remodeling-associated protein 7	**0.32 ± 0.17**	**0.33± 0.12**
**F8W7C6**	*RPL10*	60S ribosomal protein L10	**0.26 ± 0.19**	**0.33 ± 0.19**
**Q76LA1**	*CSTB*	CSTB protein	**0.29 ± 0.13**	**0.32 ± 0.19**
**Q15149-3**	*PLEC*	Isoform 3 of Plectin	**0.30 ± 0.23**	**0.30 ± 0.29**
**Q05D80**	*RNMT*	RG7MT1 (Fragment)	0.33 ± 0.28	**0.24 ± 0.25**

Proteins are ordered according to their mean abundance ratio (AR) in FLSs treated with LPC 16:0 in the presence of IL-1β. The ARs were calculated from the protein levels in treated FLSs relative to their untreated controls. Accession IDs, gene symbols, and protein names were obtained from UniProt Knowledgebase. The ARs are presented as mean ± SD, and those reproducibly regulated in at least 10 of 12 replicates are shown in bold. The mRNA expression of selected proteins was determined by RT-PCR using the $2^{-\Delta\Delta Ct}$ method. Data in brackets are presented as mean ± SD of the fold-change in mRNA expression relative to control (=1). The ARs of each protein were compared by multiple t-test, with the FDR set to 10%.

Table S3. The 147 proteins reproducibly regulated by IL-1β alone in human FLSs.

Accession ID	Gene	Protein name	AR IL-1β		
**P02795**	*MT2A*	Metallothionein-2	6.43	5.13	**
			(3.80	*1.82)*	**
**A0A024R718**	*PBEF1*	Nicotinamide phosphoribosyltransferase	5.78	2.60	**
**O60488**	*ACSL4*	Long-chain-fatty-acid--CoA ligase 4	5.06	2.15	***
**P04732**	*MT1E*	Metallothionein-1E	5.00	4.21	**
**Q6FI13**	*H2AC18; H2AC19*	Histone H2A type 2-A	4.58	2.10	***
**O43570**	*CA12*	Carbonic anhydrase 12	4.16	2.00	*
**A0A024R969**	*CHI3L1*	Chitinase-3-like protein 1	3.76	4.47	ns
**B1AP13**	*CD55*	Complement decay-accelerating factor	3.47	2.68	*
**P47712**	*PLA2G4A*	Cytosolic phospholipase A2	3.41	1.54	**
			(5.22	*2.41)*	***
**Q7Z7M4**	*SOD2*	Superoxide dismutase (Fragment)	3.36	1.35	**
			(16.1	*10.3)*	***
**A0A024R1U8**	*IGFBP4*	Insulin-like growth factor-binding protein 4	3.32	1.66	**
**Q03169**	*TNFAIP2*	Tumor necrosis factor alpha-induced protein 2	3.20	1.46	**
**P17676**	*CEBPB*	CCAAT/enhancer-binding protein beta	2.85	0.99	*
**O95379**	*TNFAIP8*	Tumor necrosis factor alpha-induced protein 8	2.81	1.13	*
**E5KRG5**	*TYMP*	Thymidine phosphorylase	2.72	0.44	**
**P02461**	*COL3A1*	Collagen alpha-1(III) chain	2.71	1.33	*
**O00469-2**	*PLOD2*	Isoform 2 of Procollagen-lysine,2-oxoglutarate 5-dioxygenase 2	2.67	1.10	*
**A0A024R8D5**		HCG30600, isoform CRA_a	2.53	1.35	*
**A0A0S2Z4X9**	*GFPT2*	Glutamine--fructose-6-phosphate transaminase (isomerizing) (Fragment)	2.47	1.11	*
**F5GZS6**	*SLC3A2*	4F2 cell-surface antigen heavy chain	2.42	0.80	*
**D6W5K2**	*TMSB10*	Thymosin, beta 10, isoform CRA_a (Fragment)	2.39	1.42	*
**Q8IWB1**	*ITPRIP*	Inositol 1,4,5-trisphosphate receptor-interacting protein	2.39	1.09	**
**P78324**	*SIRPA*	Tyrosine-protein phosphatase non-receptor type substrate 1	2.34	0.67	**
**A0A0G2JH37**	*TAPBP*	Tapasin	2.26	0.84	**
**Q04828**	*AKR1C1*	Aldo-keto reductase family 1 member C1	2.17	1.00	*
**Q9HC07**	*TMEM165*	Transmembrane protein 165	2.14	0.64	***
**Q7L7L0**	*H2AW*	Histone H2A type 3	2.12	0.67	**
			(0.75	*0.42)*	*ns*
**Q8WUJ3**	*CEMIP*	Cell migration-inducing and hyaluronan-binding protein	2.10	0.62	**
**A0A346HG00**	*HLA-A*	MHC class I antigen	2.09	0.67	*
**A0A024QZM9**	*PLAU*	Urokinase-type plasminogen activator	1.98	0.54	ns
**A0A024R6R4**	*MMP2*	72 kDa gelatinase	1.88	0.48	**
**A0A024R1M8**	*APOL2*	Apolipoprotein L, 2, isoform CRA_a	1.84	0.29	***
			(1.34	*0.88)*	*ns*

**A0A024RAV0**	*BCAT1*	Branched-chain-amino-acid aminotransferase	1.83	0.42	**
**Q14195-2**	*DPYSL3*	Isoform LCRMP-4 of Dihydro-pyrimidinase-related protein 3	1.78	0.41	*
**Q53EP0**	*FNDC3B*	Fibronectin type III domain-containing protein 3B	1.78	0.54	ns
**P07602**	*PSAP*	Prosaposin	1.74	0.40	**
**P02671**	*FGA*	Fibrinogen alpha chain	1.73	0.67	ns
**Q5H9B4**	*TIMP1*	Metalloproteinase inhibitor 1 (Fragment)	1.72<br>*(1.07*	0.39<br>*0.29)*	ns<br>ns
**Q13217**	*DNAJC3*	DnaJ homolog subfamily C member 3	1.71	0.50	***
**Q12841**	*FSTL1*	Follistatin-related protein 1	1.69	0.48	*
**P20645**	*M6PR*	Cation-dependent mannose-6-phosphate receptor	1.68	0.36	*
**P19838**	*NFKB1*	Nuclear factor NF-kappa-B p105 subunit	1.65	0.31	***
**P11498**	*PC*	Pyruvate carboxylase, mitochondrial	1.64	0.33	**
**A2ACR1**	*PSMB9*	Proteasome subunit beta	1.63	0.31	*
**A0A384NPU5**		Epididymis secretory sperm binding protein	1.62	0.21	*
**Q96KG9**	*SCYL1*	N-terminal kinase-like protein	1.58	0.37	*
**B2R4R9**	*RPS28*	40S ribosomal protein S28	1.58	0.43	*
**Q10471**	*GALNT2*	Polypeptide N-acetylgalactosaminyltransferase 2	1.58	0.42	*
**Q16851**	*UGP2*	UTP--glucose-1-phosphate uridylyltransferase	1.57	0.31	*
**V9HWB7**	*HEL60*	Citrate hydro-lyase	1.55	0.45	**
**Q92575**	*UBXN4*	UBX domain-containing protein 4	1.54	0.32	*
**N0E4C7**	*CSNK2B*	Casein kinase II subunit beta	1.54	0.45	*
**V9HWD3**	*HEL117*	Epididymis luminal protein 117	1.53	0.31	*
**Q7Z5R6**	*APBB1IP*	Amyloid beta A4 precursor protein-binding family B member 1-interacting protein	1.53	0.36	ns
**Q54A51**	*hEMMPRIN*	Basigin (Ok blood group), isoform CRA_a	1.53	0.36	*
**H0YNE9**	*RAB8B*	Ras-related protein Rab-8B (Fragment)	1.51	0.28	ns
**Q13617**	*CUL2*	Cullin-2	0.67	0.17	**
**P21926**	*CD9*	CD9 antigen	0.66	0.28	*
**O43852**	*CALU*	Calumenin	0.66	0.11	*
**P67809**	*YBX1*	Y-box-binding protein 1	0.66	0.14	ns
**Q9UHQ9**	*CYB5R1*	NADH-cytochrome b5 reductase 1	0.66	0.16	*
**Q9NQ48**	*LZTFL1*	Leucine zipper transcription factor-like protein 1	0.66	0.10	*
**Q9NVD7**	*PARVA*	Alpha-parvin	0.66	0.11	**
**A0A024R1U0**	*RANGAP1*	Ran GTPase activating protein 1, isoform CRA_d	0.65	0.24	*
**Q9HB90**	*RRAGC*	Ras-related GTP-binding protein C	0.65	0.22	*
**Q6FGY1**	*HPCAL1*	HPCAL1 protein	0.65	0.14	*
**Q8NEU8**	*APPL2*	DCC-interacting protein 13-beta	0.65	0.05	***
**Q9Y3Q8**	*TSC22D4*	TSC22 domain family protein 4	0.65	0.18	ns
**Q9ULZ3**	*PYCARD*	Apoptosis-associated speck-like protein containing a CARD	0.65	0.10	**
**Q8N8S7-2**	*ENAH*	Isoform 2 of Protein enabled homolog	0.65	0.14	*

**A0A140VJL0**		3-hydroxyisobutyryl-CoA hydrolase, mitochondrial	0.65	0.15	*
**Q6MZT2**	*DKFZp686D 1354*	Receptor protein-tyrosine kinase	0.64	0.17	*
**P13473**	*LAMP2*	Lysosome-associated membrane glycoprotein 2	0.64	0.28	ns
**Q9Y376**	*CAB39*	Calcium-binding protein 39	0.64	0.15	*
**O43181**	*NDUFS4*	NADH dehydrogenase [ubiquinone] iron-sulfur protein 4, mitochondrial	0.64	0.18	*
**Q5U043**		S-(hydroxymethyl)glutathione dehydrogenase	0.63	0.11	ns
**O15144**	*ARPC2*	Actin-related protein 2/3 complex subunit 2	0.63	0.08	**
**Q7Z4V5**	*HDGFL2*	Hepatoma-derived growth factor-related protein 2	0.63	0.12	**
**Q13813**	*SPTAN1*	Spectrin alpha chain, non-erythrocytic 1	0.63	0.05	*
**Q6SES1**	*PRNP*	Major prion protein	0.63	0.21	ns
**Q53G33**		Small nuclear ribonucleoprotein polypeptide C variant (Fragment)	0.63	0.10	**
**P60891**	*PRPS1*	Ribose-phosphate pyrophosphokinase 1	0.63	0.28	*
**D3DUZ3**	*IFI16*	Interferon, gamma-inducible protein 16, isoform CRA_a	0.62	0.20	ns
**Q9BVJ8**	*HEXA*	Beta-N-acetylhexosaminidase (Fragment)	0.62	0.10	ns
**Q59G24**		Activated RNA polymerase II transcriptional coactivator p15 (Fragment)	0.62	0.17	**
**Q96QK1**	*VPS35*	Vacuolar protein sorting-associated protein 35	0.62	0.09	**
**B7Z9B8**		cDNA FLJ56912, highly similar to Fibulin-2	0.62	0.23	ns
**O00483**	*NDUFA4*	Cytochrome c oxidase subunit NDUFA4	0.62	0.15	ns
**O95373**	*IPO7*	Importin-7	0.62	0.11	*
**P50402**	*EMD*	Emerin	0.61	0.10	**
**P62328**	*TMSB4X*	Thymosin beta-4	0.61	0.14	*
**Q13850**		Peripheral-type benzodiazepine receptor	0.60	0.18	*
**A0A024R3P9**	*ACBD3*	Acyl-Coenzyme A binding domain containing 3, isoform CRA_a	0.60	0.18	*
**A0A384MTY2**		Epididymis secretory sperm binding protein	0.60	0.06	**
**Q96AC1**	*FERMT2*	Fermitin family homolog 2	0.60	0.06	**
**A0A087WVZ9**	*POLR2E*	DNA-directed RNA polymerase II subunit E	0.59	0.16	*
**Q16531**	*DDB1*	DNA damage-binding protein 1	0.59	0.07	***
**Q01995**	*TAGLN*	Transgelin	0.59	0.11	*
**Q9P035**	*HACD3*	Very-long-chain (3R)-3-hydroxyacyl-CoA dehydratase 3	0.59	0.13	*
**P62314**	*SNRPD1*	Small nuclear ribonucleoprotein Sm D1	0.58	0.18	*
**B2R4R0**	*HIST1H4J*	Histone H4	0.58	0.19	ns
**Q6GMV3**	*PTRHD1*	Putative peptidyl-tRNA hydrolase PTRHD1	0.57	0.15	*
**Q15370**	*ELOB*	Elongin-B	0.57	0.15	**

**P62495**	*ETF1*	Eukaryotic peptide chain release factor subunit 1	0.57	0.17	**
**O00629**	*KPNA4*	Importin subunit alpha-3	0.56	0.07	**
**J3KQN4**	*RPL36A*	60S ribosomal protein L36a	0.56	0.25	*
**Q53FX5**		Lin-7 homolog C variant (Fragment)	0.56	0.29	ns
**O75821**	*EIF3G*	Eukaryotic translation initiation factor 3 subunit G	0.56	0.14	*
**B2RTQ2**	*TBC1D2B*	TBC1 domain family, member 2B, isoform CRA_b	0.56	0.24	**
**A0A384MDR3**	*FTL*	Ferritin	0.56	0.09	ns
**B4DKX4**		Programmed cell death protein 4	0.55	0.11	*
**Q6FHL3**	*TRAM1*	Translocating chain-associated membrane protein	0.55	0.10	ns
**Q5VZM2-2**	*RRAGB*	Isoform 2 of Ras-related GTP-binding protein B	0.55	0.21	*
**V9HW48**	*HEL-S-115*	SH3 domain-binding glutamic acid-rich-like protein	0.55	0.16	*
**Q8IZ07**	*ANKRD13A*	Ankyrin repeat domain-containing protein 13A	0.54	0.12	***
**Q7Z434**	*MAVS*	Mitochondrial antiviral-signaling protein	0.54	0.16	**
**E7EWW0**	*VPS35L*	VPS35 endosomal protein sorting factor-like	0.54	0.28	ns
**A0A1B0GVH5**	*FTO*	Alpha-ketoglutarate-dependent dioxygenase FTO	0.53	0.20	*
**A0A024R1A4**	*UBE2L3*	Ubiquitin-conjugating enzyme E2L 3, isoform CRA_a	0.52	0.13	**
**Q53QV2**	*LBH*	Protein LBH	0.52	0.14	**
**Q59FR8**		Galectin (Fragment)	0.51	0.16	**
**A8K644**	*SFRS4*	Splicing factor, arginine/serine-rich 4, isoform CRA_b	0.50	0.14	*
**P05114**	*HMGN1*	Non-histone chromosomal protein HMG-14	0.50	0.16	*
**P17900**	*GM2A*	Ganglioside GM2 activator	0.50	0.10	ns
**Q96FJ2**	*DYNLL2*	Dynein light chain 2, cytoplasmic	0.49	0.32	ns
**Q6NS36**	*FTH1*	Ferritin (Fragment)	0.47	0.08	ns
**P54727**	*RAD23B*	UV excision repair protein RAD23 homolog B	0.46	0.12	*
**A0A0S2Z377**	*ANXA6*	Annexin (Fragment)	0.45	0.17	*
**B8ZWD1**	*DBI*	Acyl-CoA-binding protein	0.44	0.14	*
**Q7L5D6**	*GET4*	Golgi to ER traffic protein 4 homolog	0.42	0.29	ns
**P54725**	*RAD23A*	UV excision repair protein RAD23 homolog A	0.41	0.27	*
**A8K651**		Complement component 1 Q subcomponent-binding protein, mitochondrial	0.40	0.15	**
**P09493-6**	*TPM1*	Isoform 6 of Tropomyosin alpha-1 chain	0.39	0.19	ns
**Q6IPW4**	*NDUFV2*	NADH dehydrogenase [ubiquinone] flavoprotein 2, mitochondrial	0.39	0.32	ns
**Q8NI22**	*MCFD2*	Multiple coagulation factor deficiency protein 2	0.39	0.18	**
			(0.72	*0.20)*	ns
**P37198**	*NUP62*	Nuclear pore glycoprotein p62	0.37	0.23	**
**P0DP25**	*CALM3*	Calmodulin-3	0.37	0.11	*
			(0.42	*0.09)*	***

**D3DV26**	*S100A10*	Calpactin I light chain (Fragment)	0.37	0.18	**
**B4DTX4**		cDNA FLJ59756, highly similar to Ras GTPase-activating protein 1	0.35	0.22	*
**P84157-3**	*MXRA7*	Isoform 3 of Matrix-remodeling-associated protein 7	0.32	0.17	*
**Q15149-3**	*PLEC*	Isoform 3 of Plectin	0.30	0.23	***
**Q76LA1**	*CSTB*	CSTB protein	0.29	0.13	**
**F8W7C6**	*RPL10*	60S ribosomal protein L10	0.26	0.19	***
**Q86YQ0**	*HZGJ*	HZGJ	0.22	0.13	***
**P17342**	*NPR3*	Atrial natriuretic peptide receptor 3	0.21	0.12	**
**P07951-3**	*TPM2*	Isoform 3 of Tropomyosin beta chain	0.21	0.06	**
			(0.43	*0.11)*	**
**B4DG57**	*PHF14*	PHD finger protein 14	0.18	0.14	**

Proteins are ordered according to their mean abundance ratio (AR) in IL-1β-treated FLSs. Accession IDs, gene symbols, and protein names were obtained from UniProt Knowledgebase. The ARs were calculated from the protein levels in IL-1β-treated FLSs relative to their untreated control. The ARs are presented as mean ± SD. The mRNA expression of selected proteins was determined by RT-PCR using the $2^{-\Delta\Delta Ct}$ method. Data in brackets are presented as mean ± SD of the fold-change mRNA expression relative to control (normalized to 1). *0.05 > P > 0.01; **0.01 > P >0.001; ***P < 0.001; ns: P > 0.05.
